# Assessing Patient Confidence and Satisfaction about the Shared Decision-making Meetings for Planning Cancer Chemotherapy

**DOI:** 10.7759/cureus.6445

**Published:** 2019-12-22

**Authors:** Shadi S Alkhayyat, Hussain Hudairi, Rakan M Alqahtani, Waleed Alqulayti, Abdulelah Kinkar, Mohannad Alghamdi, Shihab Alhakami

**Affiliations:** 1 Oncology, King Abdulaziz University Hospital, Jeddah, SAU; 2 Internal Medicine, King Abdulaziz University Hospital, Jeddah, SAU

**Keywords:** cancer chemotherapy planning, decision sharing, patients, satisfaction

## Abstract

Background

Patients increasingly express the desire to be involved in their treatment decisions, especially in critical situations, such as cancer chemotherapy that increase a doctor's responsibility toward fulfilling these needs. This process may require more than one meeting with the patient to meet their expectations and satisfaction levels. This study aimed to assess the satisfaction levels in cancer patients, who received chemotherapy, about their decision-making and if they were able to make this decision during the first meeting with their physicians.

Methods

A cross-sectional study was conducted in 2017 on 106 cancer patients aged 18 years or above who were receiving chemotherapy at the day-care unit of King Abdulaziz University Hospital (KAUH), Jeddah, Saudi Arabia. The data were collected by a direct or telephonic interview using a structured questionnaire. The variables were studied across two groups of patients based on the patient's ability to make decision in the first meeting with their physician. Data were expressed as frequencies (percentage) and Pearson Chi-Square test was used to assess the categorical variables.

Results

Out of the 106 patients, 42 (39.6%) of them were male. Ninety-one (85.8%) patients took the decision by themselves. Regarding the decision-making 90 (84.9%) patients were able to make the decision from the first meeting. Sixty-eight (64.2%) patients felt more satisfied if they had an additional session. There was a significant association between patients with the ability to make the decision during the first meeting and patients who took the decision by themselves (P = 0.033), patients with consideration of changing their decision if they had more meetings (P = 0.005), patients with consideration of withholding from chemotherapy in their mind (P = 0.019) and patients with thought that chemotherapy is affecting their life (P = 0.044).

Conclusion

The majority of the patients felt that more than one meeting with their doctors would be helpful in improving their satisfaction level during the decision-making process, consideration of withholding from chemotherapy in mind and that chemotherapy is affecting their life style. Future protocol in which the patients will be encouraged to have a confidence role on their treatment decision is recommended.

## Introduction

Cancer is a major healthcare challenge worldwide, and it is one of the leading causes of deaths, accounting for 9.6 million deaths in 2018. Lung cancer is associated with the highest incidence of cancer-related deaths [1]. In Saudi Arabia, cancer ranked tenth among the causes of death in 2015, and colorectal cancer and breast cancer are the commonest cancers among males and females, respectively [2].

Cancer treatment is usually multidisciplinary, involving a medical oncologist, surgical oncologist, and radiation oncologist [3]. Chemotherapy is commonly used to treat cancer patients, both as a curative or palliative therapy to extend survival, improve the quality of life, and delay critical complications [4]. Chemotherapy is a high-risk medication that can be associated with short-term complications including, nausea, vomiting, fatigue, pain, numbness, or alopecia, and long-term complications, including memory problem, fertility issue, secondary malignancy, or nerve damage [5-7].

Over the past few years, patients are increasingly demanding more information as the access to information through the Internet is improving [8,9]. This is especially relevant in critical illnesses where most of the patients feel unsure regarding decision-making [10,11]. Further, the decision, whether to receive chemotherapy, is highly influenced by the historical concept of the side effects of chemotherapy and to a lesser extent, by the poor concept of palliative care [12,13]. Providing the patients with accurate information about the benefits and complications of chemotherapy will help their decision-making and hence, improve patient compliance to chemotherapy and acceptance of possible side effects [14,15]. Because of these challenges in the process of decision-making about chemotherapy, we were interested in assessing the patient confidence in the current decision-making process and if having more than one session of discussion with the physicians would help to improve the patient confidence in the process of decision-making.

This questionnaire-based study aimed to assess the satisfaction levels in cancer patients, who received chemotherapy, about their decision-making and if they were able to make this decision during the first meeting with their physicians.

## Materials and methods

This questionnaire-based cross-sectional study was conducted in the Day Care Unit at King Abdulaziz University Hospital (KAUH), Jeddah, Saudi Arabia from January to March, 2017. All patients aged 18 years or above who received chemotherapy during the study period were included, resulting in enrollment of 106 patients. This study was approved by the Research Ethics Committee (REC) of King Abdulaziz University, Jeddah, Saudi Arabia and was made according to principles of Helsinki Declaration.

Due to the lack of a pre-validated tool, a formulated electronic questionnaire was formatted based on previous studies, as shown in Table 1. The questionnaire was prepared in English and translated into Arabic by well-trained medical students, and it was reviewed by oncology expert (professor and consultant). The validity of the questionnaire was established by face validity through consultation with a panel of experts in oncology field. The reliability of questionnaire was evaluated using Cronbach's alpha index by SPSS software. Value more than 0.7 shows questionnaire reliability. All data were obtained from each enrolled patient by direct and telephone interview using a questionnaire only after they received a full explanation of the purpose of this study and gave their verbal consent.

**Table 1:** Electronic questionnaire DM: Diabetes mellitus; HTN: Hypertension; IHD: Ischemic heart disease.

Data collection sheet											
Q1. Do you agree for participating on this questionnaire?											
Yes						No					
Q2. Nationality?											
Saudi						None-Saudi					
Q3. How old are you?											
less than 50	50-60			61-70		71-80		81-90			91-100
Q4. Gender?											
Male						Female					
Q5. What is your education level?											
Illiterate			School			Bachelors			Master or higher		
Q6. What type of cancers do you have?											
Breast cancer				Colorectal cancer				Others			
Q7. Do you have any chronic diseases?											
Yes e.g.: (DM, HTN, IHD, Hyperlipidemia)						No					
Q8. Do you take any chronic medication other than chemotherapy?											
Yes						No					
Q9. Was there any language barrier, during or after the meeting about chemotherapy decision?											
Yes						No					
Q10. How long have you been diagnosed with cancer?											
3-6 months			7-9 months			10-12 months			More than 12 months		
Q11. When did you start the course of chemotherapy?											
3-6 weeks			7-9 weeks			10-12 weeks			More than 12 weeks		
Q12. Were you the one who took the decision about chemotherapy?											
Yes						No					
Q13. Were you able to make this decision from the first meeting with your doctor?											
Yes						No					
Q14. Do you think if you have more than one meeting, your decision would be changed?											
Yes						No					
Q15. Did you feel satisfied about the information you received from your physician to make the decision to start chemotherapy?											
Satisfied						Unsatisfied					
Q (16-17) Related to each other.											
Q16. Do you think having additional meetings with your physician will improve your satisfaction about the decision?											
Yes						No					
Q17. How much this will improve the satisfaction?											
Unsatisfied		Natural			Satisfied		Very satisfied			Very unsatisfied	
Q18. What was the purpose of the chemotherapy?											
Curative				Palliative				Patient doesn’t know			
Q19. Have you ever considered withholding from chemotherapy in your mind?											
Yes						No					
Q20. How much did the chemotherapy affect your life/lifestyle?											
Has no effect at all				A little bit effect				Great effect			

The questionnaire was formed of 20 questions that assess demographic and clinical characteristics and patient's opinion about sharing in chemotherapy decision. Demographic data such as age, gender, and current level of education were collected. Following clinical characteristics were obtained from each enrolled patient as presence of any coexisting chronic diseases, current prescription medications for chronic diseases, time elapsed since the diagnosis of cancer, duration of chemotherapy, purpose of chemotherapy, side effects of chemotherapy leading to contemplation or actual discontinuation of chemotherapy, and the impact of chemotherapy on their life.

Patients' satisfaction with the information that had been provided by their physicians in the meeting was assessed. Further, the patients were asked about the following factors that may have influenced their decision-making about chemotherapy: if they took the decision by themselves, if provision of an additional meeting with their physician would change their decision and/or improve their confidence about the decision, and if they faced any language barriers during the meeting with their physicians. Data entry was performed by using Microsoft Excel 2016.

Statistical analysis

Data were analyzed using IBM SPSS Statistics for Windows, version 21 (IBM Corp., Armonk, NY). Descriptive statistics were reported as frequencies and percentages for categorical variables. The patients were categorized into two groups based on ability to make decision in the first meeting with the physician. Inter-group differences were examined using the Pearson Chi-Square test. P-values less than 0.05 were considered as significant.

## Results

In this cross-sectional study, 106 patients participated, out of them the most age group was <50 years (n = 41, 38.7%) and least age group was 81-90 years (n = 2, 1.9%). The males (n = 42, 39.6%) were lower than females (n = 64, 60.4%). The non-Saudi participants (n = 69, 65.1%) were more than Saudi (n = 37, 34.9%). The education levels were mostly school (43.4%) then illiterate (n = 30, 28.3%), bachelors (n = 25, 23.6%) and lastly masters or higher (n = 5, 4.7%). Chronic diseases were reported in 55 (51.9%), while 55 (51.9%) received medications and only 19 (18%) had a language barrier. The most common cancer reported among our participants was breast cancer (n = 48, 45.3%), followed by colorectal (n = 18, 17.0%) and others (37.7%). Length of cancer diagnosis and start of chemotherapy were mostly more than 12 months (42.5% and 46.2%, respectively) (Table 2).

**Table 2:** Demographic and clinical characteristics of study participants

Category		Number (%)
Age groups	<50 years	41 (38.7%)
	50-60 years	37 (34.9%)
	61-70 years	22 (20.8%)
	71-80 years	4 (3.8%)
	81-90 years	2 (1.9%)
Gender	Male	42 (39.6%)
	Female	64 (60.4%)
Nationality	Saudi	37 (34.9%)
	Non-Saudi	69 (65.1%)
Education level	Illiterate	30 (28.3%)
	School	46 (43.4%)
	Bachelors	25 (23.6%)
	Masters or higher	5 (4.7%)
Chronic diseases	Yes	54 (50.9%)
	No	52 (49.1%)
Medications	Yes	55 (51.9%)
	No	51 (48.1%)
Patient had a language barrier	Yes	19 (18%)
	No	87 (82.0%)
Type of cancer	Breast cancer	48 (45.3%)
	Colorectal cancer	18 (17.0%)
	Others	40 (37.7%)
Length of cancer diagnosed	3-6 months	39 (36.8%)
	7-9 months	11 (10.4%)
	10-12 months	11 (10.4%)
	More than 12 months	45 (42.5%)
Start of chemotherapy course	3-6 months	35 (33.0%)
	7-9 months	11 (10.4%)
	10-12 months	11 (10.4%)
	More than 12 months	49 (46.2%)

Ninety (84.9%) patients were able to make the decision during the first meeting, and 16 (15.1%) patients required additional meetings with the doctor. Table 3 showed the results for factors affecting the decision-making process. Ninety-one (85.8%) patients took the decision by themselves, while 15 (14.2%) didn't. Seventy-two patients (67.9%) did not think that their decision about chemotherapy would be changed if they had additional meetings. The majority of patients, i.e. 88 (83%), were satisfied with the information provided by their doctors, while only 18 (17%) were not satisfied. However, most of the patients, i.e. 68 (64.2%), thought additional sessions would further increase their satisfaction levels. Eighty-eight (83.0%) patients used chemotherapy for curative, 12 (11.3%) patients did not know the purpose, and only six (5.7%) reported using chemotherapy for palliative care. Thirty-three (31.1%) patients thought about withholding the chemotherapy course. On opinion of the patients about chemotherapy effects on life, most patients (48.1%) reported that chemotherapy had great effect, while 38.7% reported that it had little bit effect and 13.2% reported that it had no effect. There were significant associations between the patients with the ability to make the decision during the first meeting and the patients who took the decision by themselves (P = 0.033), with consideration of changing their decision if they had more meetings (P = 0.005), with consideration of withholding from chemotherapy in their mind (P = 0.019) and with those chemotherapy affecting their life (P = 0.044).

**Table 3:** Factors that influence decision making

Items		Total (n = 106)	Patient was able to make the decision during the 1st meeting		P-value
			Yes (n = 90, 84.9%)	No (n = 16, 15.1%)	
Patient took the decision by:	Themselves	91 (85.8%)	80 (88.9%)	11 (68.8%)	0.033*
	Others	15 (14.2%)	10 (11.1%)	5 (31.2%)	
Patient thought that having additional meeting will change his/her decision	Yes	34 (32.1%)	24 (26.7%)	10 (62.5%)	0.005**
	No	72 (67.9%)	66 (73.3%)	6 (37.5%)	
Patient satisfaction about the information given by doctor	Satisfied	88 (83.0%)	75 (83.3%)	13 (81.3%)	0.838
	Unsatisfied	18 (17.0%)	15 (16.7%)	3 (18.7%)	
Patient thought that having additional meeting will improve satisfaction	No	38 (35.8%)	34 (37.8%)	4 (25.0%)	0.326
	Yes	68 (64.2%)	56 (62.2%)	12 (75.0%)	
Improve of satisfaction	Neutral	41 (38.7%)	36 (40.0%)	5 (31.2%)	0.609
	Satisfied	33 (31.1%)	26 (28.9%)	7 (43.8%)	
	Unsatisfied	4 (3.8%)	4 (4.4%)	-	
	Very satisfied	24 (22.6%)	20 (22.2%)	4 (25.0%)	
	Very unsatisfied	4 (3.8%)	4 (4.4%)	-	
Purpose of the chemotherapy	Curative	88 (83.0%)	74 (82.2%)	14 (87.5%)	0.785
	Palliative	6 (5.7%)	5 (5.6%)	1 (6.2%)	
	Patient doesn’t know	12 (11.3%)	11 (12.2%)	1 (6.2%)	
Consider withholding from chemotherapy in your mind	Yes	33 (31.1%)	24 (26.7%)	9 (56.2%)	0.019*
	No	73 (68.9%)	66 (73.3%)	7 (43.8%)	
Chemotherapy affects life/lifestyle	A little bit effect	41 (38.7%)	37 (41.1%)	4 (25.0%)	0.044*
	Great effect	51 (48.1%)	39 (43.3%)	12 (75.0%)	
	Has no effect at all	14 (13.2%)	14 (15.6%)	-	

## Discussion

This study was conducted to assess the patient satisfaction and their preferences related to the decision-making process about cancer chemotherapy. The study also aimed to identify if the patients can make the decision during the first meeting with the physicians or if they needed additional meetings to make the decision. Majority of patients involved in this study were more than 50 years old and most of them were females with breast cancer, while majority of males in our sample had colorectal cancer, which is representative of the Saudi Arabia’s population [2].

The decision to accept chemotherapy is quite important. Patients need to understand the advantages and disadvantages of chemotherapy before they decide the future course. The time required for discussion and the ability of patient to assimilate complex information, and hence, to make informed decision is different from one patient to another. In this study, majority of patients (n = 68; 64.2%) indicated that they would be more satisfied with the decision-making if they had additional session with their physician. They believed that additional discussion about the chemotherapy would increase their confidence in making such a decision. In addition, previous study showed that the more the time spent with the patient, the more the psychological support they have while making such important decision [16]. However, in this study 90 (84.9%) patients were able to make their decision during the first meeting. Eighty-eight (83.0%) patients were satisfied about the information provided by the treating physician in the clinic from the first visit. Furthermore, 34 (32.1%) patients were convinced that additional meeting will affect their decision. Although, the results obtained by this study support that an additional meeting would improve the willingness of the patients in deciding in support of chemotherapy and improve their satisfaction, however, it will not be of great significance in altering overall acceptance rates of chemotherapy, as most patients understand that chemotherapy would enhance their survival chances in the first meeting. Establishing communication between patients and physicians that provides support and education, institute effective treatment strategy, and provides effective follow-up with patients, all these will improve patient adherence rates to chemotherapy and patients outcomes [17].

The limitations of the study include language barrier for some patients, and small sample size due to the short period of data collection. Importantly, our sample did not include patients who declined chemotherapy, if such patients were included, the impact of an additional meeting with them might affect their decision. However, the number of such patients is limited, as most of patients do believe that chemotherapy would enhance their survival.

## Conclusions

From the results of this study we can conclude that most of our participants were aged more than 50 years, and most of them were females with breast cancer who received chemotherapy for long duration. Most patients thought that if they had additional meetings with their doctors, it would be helpful in improving their satisfaction level. Additionally, this would provide them with adequate information and improve their involvement in shared decision-making process. There were significant associations between patients with the ability to make the decision during the first meeting and patients with consideration of changing their decision if they had more meetings with their physicians, patients with consideration of withholding from chemotherapy in their mind and also patients with thought that chemotherapy is affecting their life.
